# Effects of n-3 polyunsaturated fatty acid on metabolic status in women with polycystic ovary syndrome: a meta-analysis of randomized controlled trials

**DOI:** 10.1186/s13048-023-01130-4

**Published:** 2023-03-17

**Authors:** Jie Zhou, Wenting Zuo, Yong Tan, Xudong Wang, Meihong Zhu, Huili Zhang

**Affiliations:** 1 Nanjing University of Chinese Medicine, Nanjing, 210023 People’s Republic of China; 2 Department of Chinese Medicine, The First People’s Hospital of Nantong, Nantong, 226001 Jiangsu People’s Republic of China; 3 Department of Reproductive Medicine, Affiliated Hospital of Nanjing University of Chinese Medicine, Nanjing, 210029 Jiangsu People’s Republic of China; 4 Department of Pharmacy, The First People’s Hospital of Nantong, Nantong, 226001 Jiangsu People’s Republic of China; 5 Medical Department, The First People’s Hospital of Nantong, Nantong, 226001 Jiangsu People’s Republic of China

**Keywords:** n-3 polyunsaturated fatty acid, Polycystic ovary syndrome, Metabolic status, Meta-analysis, Randomized controlled trials

## Abstract

This meta-analysis was conducted to summarize the effects of *n*-3 polyunsaturated fatty acid (n-3 PUFA) on metabolic status including insulin metabolism and lipid metabolism in women with polycystic ovary syndrome (PCOS) by randomized controlled trials (RCTs). Four mainstream databases including PubMed, Cochrane Library, Embase and Web of Science were searched from their inception to October 2021. The registration number of this study was CRD42021285233. The quality assessment was performed referring the Cochrane Risk of Bias Tool. Mean differences (MD) and 95% confidence intervals (CIs) were generated for continuous variables by meta-analysis. Subgroup analyses were performed based on study duration (≤ 8 weeks or > 8 weeks), the source of n-3 PUFA (marine derived or plant origins) and dosage of n-3 PUFA (≤ 1000 mg/d or > 1000 mg/d). Eventually, 11 RCTs reporting 816 patients were enrolled. Compared with control group, n-3 PUFA treatment decreased waist circumference (MD = -2.76, 95% CI: -3.82 to -1.69; *p* < 0.00001), fasting plasma glucose (MD = -3.91, 95% CI: -5.69 to -2.13; *p* < 0.0001), fasting insulin (MD = -2.45, 95% CI: -3.19 to -1.71; *p* < 0.00001), homeostatic model assessment of insulin resistance (MD = -0.45, 95% CI: -0.80 to -0.11; *p* = 0.01), triglyceride (MD = -9.33, 95% CI: -10.56 to -8.10; *p* < 0.00001), total cholesterol (MD = -12.32, 95% CI: -19.15 to -5.50; *p* = 0.0004), low-density lipoprotein cholesterol (MD = -10.53, 95% CI: -19.31 to -1.75; *p* = 0.02), and increase quantitative insulin sensitivity check index (MD = 0.01, 95% CI: 0.01 to 0.02; *p* < 0.00001), Adiponectin (MD = 1.46, 95% CI: 1.12 to 1.80; *p* < 0.00001) in PCOS patients. However, n-3 PUFA failed to change body weight, body mass index, high-density lipoprotein cholesterol, very low-density lipoprotein cholesterol and hs-CRP in the overall analysis. Further subgroup analyses showed that supplements of n-3 PUFA for more than 8 weeks is more conducive to improve the metabolic status in insulin resistance and lipid profiles. The meta-analysis demonstrates that n-3 PUFA may be an effective intervention for alleviating metabolic status in PCOS. Hence, we recommend PCOS patients replenish n-3 PUFA with duration > 8 weeks regardless of the source and the dosage to retard the pathogenesis of PCOS related metabolic diseases.

**Supplementary Information:**

The online version contains supplementary material available at 10.1186/s13048-023-01130-4.

## Introduction

Polycystic ovary syndrome (PCOS) is a complicated reproductive endocrine disease, which can affect women’s reproductive, metabolic, and mental health [1]. It is predominantly manifested as hirsutism, menstrual disorder, hyperandrogenism and infertility and insulin resistance (IR). It also increases the risk for dyslipidemia, type 2 diabetes, obesity and cardiovascular disease [2, 3]. Approximately 50–70% of women with PCOS accompanied with IR may develop metabolic syndrome, which will also enhance the risk of other chronic diseases in the future [2–6]. In worldwide reports, the prevalence of PCOS ranges from 4 to 21% depending on different study population and diagnostic criteria [3]. A recent meta-analysis involving 154,599 Chinese participants reported that the prevalence of PCOS in China was as high as 10.01% [7].

At present, it is considered that lifestyle modification, pharmacological treatments and nutritional supplements are beneficial to PCOS [8, 9]. Because of the possible side effects of pharmacological treatments in PCOS patients, more attentions have been paid to nutritional supplements [10]. N-3 polyunsaturated fatty acid (n-3 PUFA), as a kind of nutritional supplements, has been verified to participate in the development of multiple metabolism-related diseases, including PCOS [11, 12].

International guidelines have recommended that the general public should consume 250 mg/day of n-3 PUFA [13]. Evidence from a number of studies showed that n-3 PUFA was beneficial to patients with cardiovascular disease and could decrease the risk of cardiac death [14, 15]. In addition, n-3 PUFA could reduce the blood pressure of untreated hypertensive and normotensive subjects by reducing oxidative stress, altering the function of membrane associated proteins and regulating the release of vasodilators [14]. Moreover, n-3 PUFA is thought to improve IR by regulating mitochondrial bioenergy and endoplasmic reticulum stress [16]. Some studies also showed that n-3 PUFA played an important role in changing serum lipid profile and membrane lipid composition, and affecting cell signal cascade and gene expression [17, 18].

Recently, investigators also demonstrated that n-3 PUFA had a beneficial effect on the metabolism and health of women with PCOS [19–21]. One study found that n-3 PUFA reduced total testosterone levels, IR, inflammatory cytokines and inhibited oxidative stress in patients with PCOS [20]. Another double-blind clinical trial indicated that n-3 PUFA improved lipid profiles and decreased waist circumferences (WC) in them [19]. A randomized controlled trial (RCT) comprising 60 patients also showed that flaxseed oil n-3 PUFA for 12 weeks significantly ameliorated insulin metabolism, mF-G scores, serum triglycerides (TG), cholesterol and high sensitivity C-reactive protein (hsCRP) levels in PCOS [21]. However, a previous meta-analysis including three RCTs with 72 cases and 73 controls indicated that supplementation of n-3 PUFA might not relieve IR in women with PCOS [22].

Although given the divergent published articles, as far as we know, there was no meta-analysis assessing n-3 PUFA effects on metabolic status in PCOS with subgroup analyses based on study duration, the sources and the dosage of n-3 PUFA. Therefore, the objective of this meta-analysis was to systematically evaluate the efficacy of n-3 PUFA supplementation on metabolic status in patients with PCOS.

## Methods

### Study design

We performed the study in accordance with Preferred Reporting Items for Systematic Reviews and Meta-Analysis (PRISMA) guidelines [23]. And our research protocol had been registered on PROSPERO (CRD42021285233).

### Data sources and searches

Four databases including PubMed, Cochrane library, Embase and Web of science were searched from their inception to October 2021 according to the established search strategies composed of medical subject headings (MeSH) or synonym. There were no restrictions set on the publication date, study design and language. The search results were imported into the bibliographic management tool named Endnote software. Table S1 showed the details of search strategies in PubMed.

### Study selection

#### Inclusion criteria

1. Participants: adult females who were diagnosed with PCOS according to Rotterdam 2003 criteria [24] or National Institute of Health (NIH) criteria [25] or the specialists consensus [26, 27];
2. Intervention: use of n-3 PUFA supplements or foods containing sufficient n-3 PUFA(at least 1000 mg/d) for at least 8 weeks without limitation of the source;
3. Comparison: use of placebo or foods not containing n-3 PUFA;
4. Outcomes: including at least one of following statistics, namely the metabolic status in IR (fasting plasma glucose (FPG), fasting Insulin (FINS), homeostatic model assessment-insulin resistance (HOMA-IR), quantitative insulin sensitivity check index (QUICKI), Adiponectin), the metabolic status in lipid profiles (TG, total cholesterol (TC), high-density lipoprotein cholesterol (HDL-C), low-density lipoprotein cholesterol (LDL-C), very low density lipoprotein (VLDL-C), hs-CRP and anthropometric indices (body weight (BW), body mass index (BMI), WC);
5. Study design: RCT.

#### Exclusion criteria

1. Women who were pregnant, smoking, postmenopause, with uncontrolled hypertension (> 160/100 mmHg), uncontrolled thyroid disease, androgen-secreting tumor, liver disease, Cushing syndrome or hyperprolactinemia;
2. Use of n-3 PUFA supplements or foods containing n-3 PUFA within the past 3 months before the oneset of PCOS;
3. Animal experiments, reviews, case reports, unable to obtain full-text articles or unavailable data.

### Quality assessment and Data extraction

Two investigators independently assessed the quality and extracted data of all included studies. The quality assessment was performed referring the Cochrane Collaboration’s tool.

The following data were collected from enrolled studies: the first author’s name and country, study publication year, study duration of follow-up, the sample sizes in intervention and control groups, study design; patients’ BW, BMI, WC and outcomes of the metabolic status in IR (FPG, FINS, HOMA-IR, QUICKI, Adiponectin), and the metabolic status in lipid profiles (TG, TC, HDL-C, LDL-C and VLDL-C) hs-CRP. Any discrepancy was adjudicated by a senior investigator.

### Statistical analyses

All statistical analyses were conducted by using Review Manager, version 5.2. A value of *p* < 0.05 was considered to be statistically significant. Mean differences (MD) and 95% confidence intervals (CIs) were generated for continuous variables by meta-analysis. If I^2^ < 50% and Q-test *p* > 0.01, then a fixed effect model would be used, otherwise a random effect model would be implemented. Heterogeneity was assessed as low (I^2^ ≤ 50%), moderate (50% < I^2^ ≤ 75%), and high (I^2^ > 75%). If I^2^ > 50%, further analysis encompassing sensitivity analysis or subgroup analysis might be carried out to explore the source of heterogeneity. Subgroup analyses were performed based on study duration (≤ 8 weeks or > 8 weeks), sources of n-3 PUFA (marine derived or plant origins) and dosage of n-3 PUFA (≤ 1000 mg/d or > 1000 mg/d). Funnel plots were used to evaluate publication bias.

### Certainty assessment

Grading of Recommendations Assessment, Development and Evaluation (GRADE) approach [28] was used to assess the quality of evidence for each indicator. Five factors were considered in the overall quality evaluation: risk of bias, inconsistency, indirectness, imprecision and publication bias. he quality of evidence was downgraded one level when each factor was accomplished. Overall quality of evidence levels were classed into high, moderate, low or very low quality.

## Results

### Study selection and Study characteristics

**Fig. 1:**
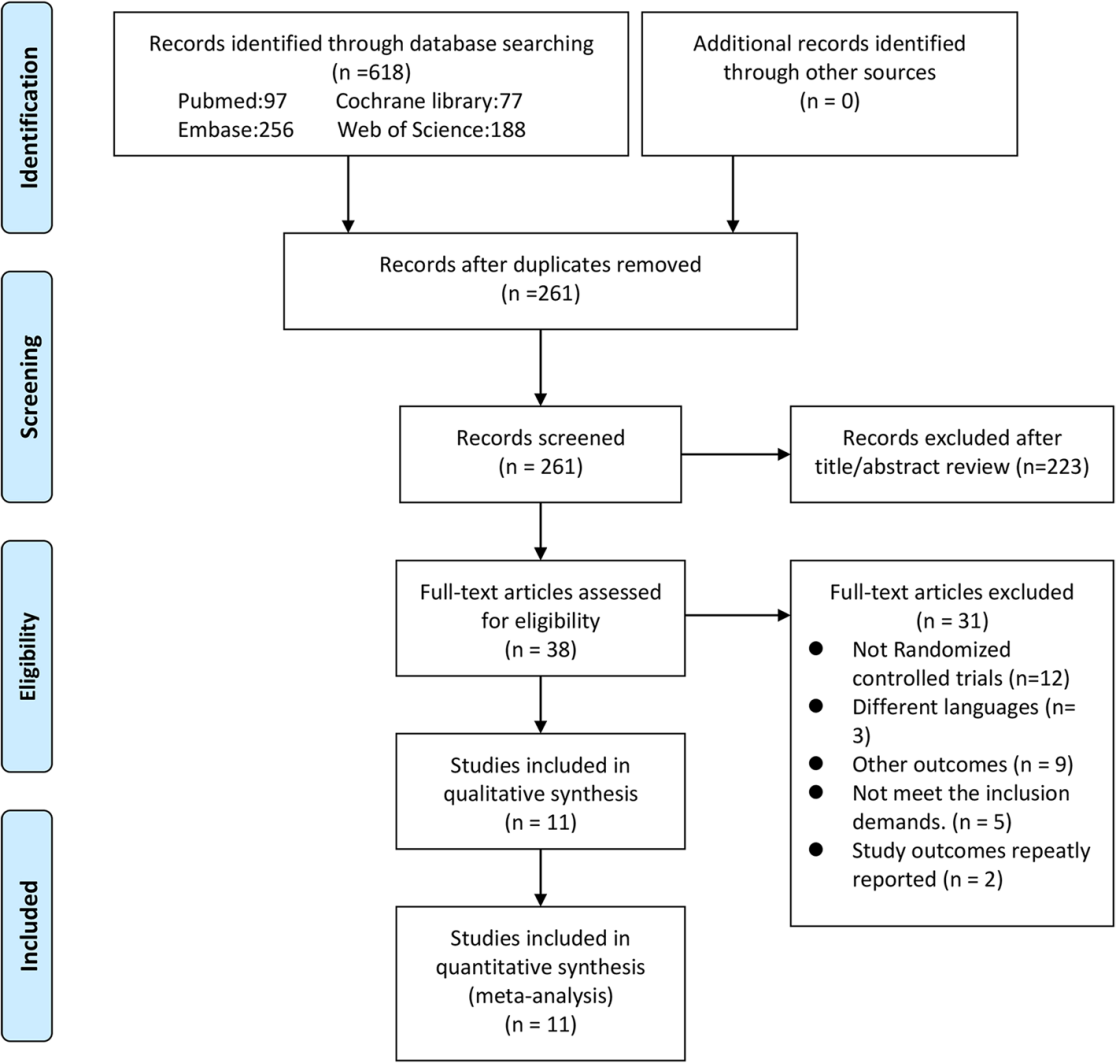
PRISMA flow diagram of study selection process

Figure 1 showed the screening procedure. Eventually, 11 RCTs [19–21, 26, 29–35] reporting 816 patients were enrolled, as shown in Table 1. Patients were allocated into two groups: n-3 PUFA group and control group. These studies were conducted in different countries, ie. 1 in Australia, 1 in Venezuela and 9 in Iran (with 4 of the Iranian studies coming from the same research group of Asemi). In all studies, the control groups were treated with certain oil comprising paraffin oil, soybean oil or olive oil capsules. Study duration of all the studies ranged from 8 to 24 weeks. The sources of n-3 PUFA mainly included marine derived (fish oil, docosahexaenoic acid (DHA) and eicosapentaenoic acid (EPA)) and plant origin (flaxseed oil). The dosage of n-3 PUFA ranged from 1000 to 4000 mg/d. Most of the patients in these studies were diagnosed with PCOS according to the Rotterdam criteria. Only one study referred NIH criteria and one study referred the specialists consensus. One study was conducted in Oceania, one in South America, and nine in the Middle East. Specific indicators and values involved in all included studies were listed in Table S2.

**Table 1:** The characteristics of included studies

Study	Country	Study design	Diagnostic criteria	Sample size		Diet type		Study duration (weeks)	Outcomes
				Intervention group	Control group	Intervention group	Control group		
Amini 2018 [20]	Iran	Randomized, double blinded	Rotterdam criteria	27	27	Fish oil n-3 PUFA supplements 2000 mg/d (Marine derived)	Paraffin oil (placebo) 100 mg/d	12	BW,BMI,hs-CRP,QUICK,FPG, FINS, HOMA-IR, TG, TC, LDL-C, HDL-C,VLDL-C
Cussons 2009 [32]	Australia	Randomized, double blinded	Rotterdam criteria	12	13	Fish oil n-3 PUFA 4000 mg/d (Marine derived)	olive oil (placebo) 4000 mg/d	8	BMI,hs-CRP,FPG, FINS, HOMA-IR, TG, TC, LDL-C, HDL-C
Ebrahimi 2017 [30]	Iran	Randomized, double blinded	Rotterdam criteria	34	34	Flaxseed oil n-3 PUFA 1000 mg/d (Plant origins) + vitamin E 400 IU/d	Placebo	12	BW,BMI,QUICK,FPG, FINS, HOMA-IR
Jamilian 2018 [33]	Iran	Randomized, double blinded	Rotterdam criteria	30	30	Fish oil n-3 PUFA 2000 mg/d (Marine derived) + vitamin E 50,000 IU/2 weeks	Placebo	12	BMI, BW, hs-CRP
Khani 2017 [19]	Iran	Randomized, double blinded	NIH criteria	43	44	n-3 PUFA supplements 2000 mg/d (360 mg EPA and 240 mg DHA) (Marine derived)	Olive oil (placebo) 2000 mg/d	24	BMI,WC,FPG, TG, TC, LDL-C, HDL-C
Mejia-Montilla 2018 [26]	Venezuela	Randomized, double blinded	Specialists Consensus	97	98	n-3 PUFA supplements 1000 mg/d (180 mg EPA and 120 mg DHA)	Paraffin oil (placebo) 1000 mg/d	12	BMI,FINS, HOMA-IR, TG, TC, LDL-C, HDL-C,Adiponectin
Mirmasoumi 2018 [21]	Iran	Randomized, double blinded	Rotterdam criteria	30	30	Flaxseed oil n-3 PUFA 2000 mg/d (Plant origins)	Paraffin oil (placebo) 500 mg/d	12	BW,BMI,hs-CRP,QUICK,FPG, FINS, HOMA-IR, TG, TC, LDL-C, HDL-C,VLDL-C
Mohammadi 2012 [31]	Iran	Randomized, double blinded	Rotterdam criteria	30	31	n-3 PUFA supplements 4000 mg/d (720 mg EPA and 480 mg DHA) (Marine derived)	Paraffin oil (placebo) 500 mg/d	8	BW,BMI,WC,hs-CRP,FPG, FINS, HOMA-IR, TG, TC, LDL-C, HDL-C,Adiponectin
Nadjarzadeh 2015 [34]	Iran	Randomized, double blinded	Rotterdam criteria	39	39	n-3 PUFA supplements 1000 mg/d (180 mg EPA and 120 mg DHA)	Paraffin oil (placebo) 1000 mg/d	12	BMI,WC,Adiponectin
Rahmani 2017 [29]	Iran	Randomized, double blinded	Rotterdam criteria	34	34	Flaxseed oil n-3 PUFA 1000 mg/d (Plant origins) + Vitamin E 400 IU/d	Placebo	12	BW,BMI,TG, TC, LDL-C, HDL-C,VLDL-C
Talari 2018 [35]	Iran	Randomized, double blinded	Rotterdam criteria	30	30	Flaxseed oil n-3 PUFA 1000 mg/d (Plant origins) + Vitamin E 400 IU/d	Placebo	12	hs-CRP

*Abbreviations: BW* body weight, *BMI* body mass index, *DHA* docosahexaenoic acid, *EPA* eicosapentaenoic acid, *FPG* fasting plasma glucose, *FINS* fasting insulin, *HDL-C* high-density lipoprotein cholesterol, *HOMA-IR* homeostatic model of assessment for insulin resistance, *hs-CRP* high sensitivity C-reactive protein, *LDL-C* low-density lipoprotein cholesterol, *n-3 PUFA* n-3 polyunsaturated fatty acid, *QUICK* quantitative insulin sensitivity check index, *TC* total cholesterol, *TG* triglycerides, *VLDL-C* very low density lipoprotein-cholesterol

### Risk of bias within studies

**Fig. 2:**
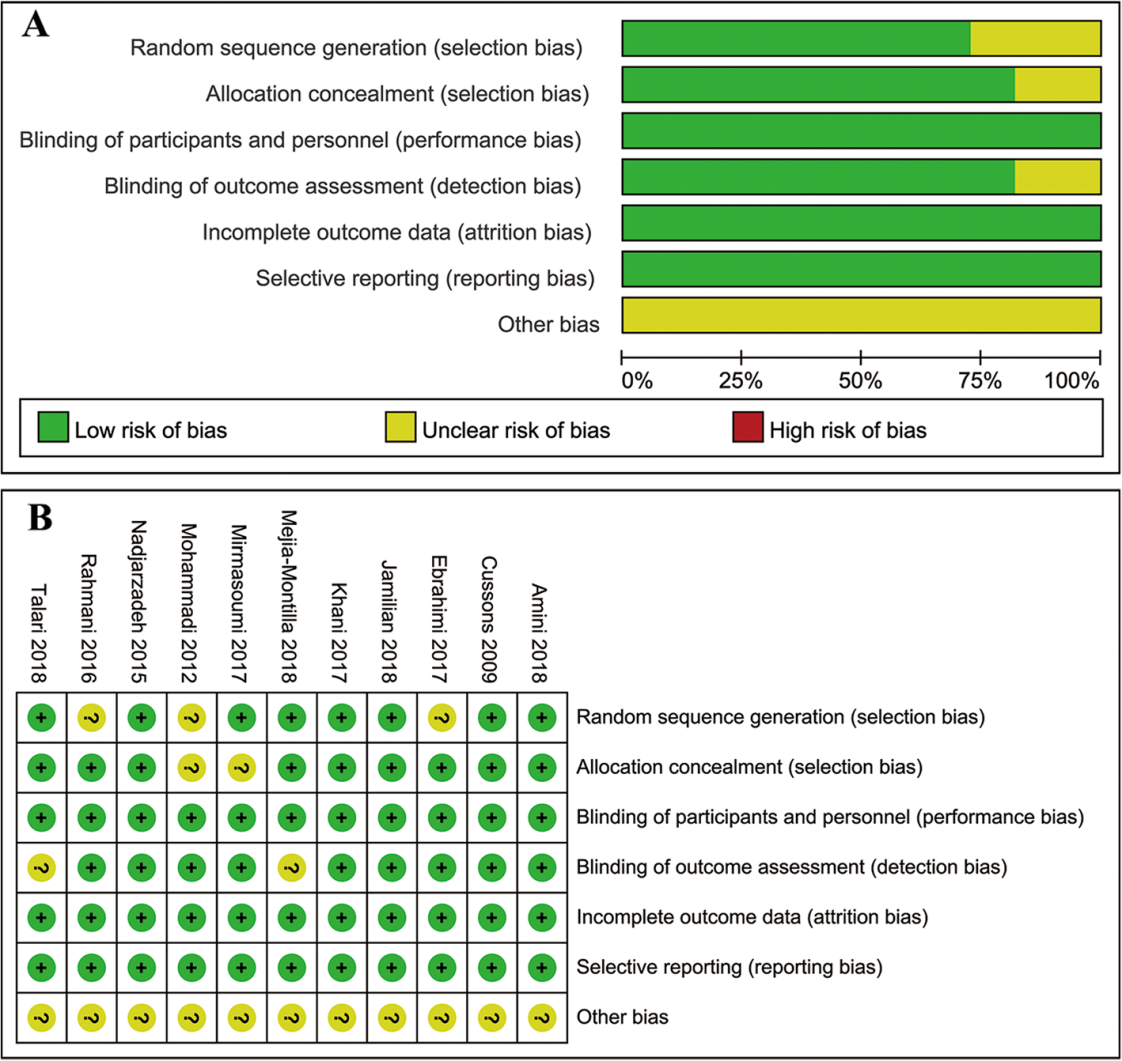
Risk of bias graph of RCTs (**A**); Risk of bias summary of RCTs (**B**)

The results of assessment of risk of bias across studies were shown in Fig. 2. All included studies reported the method of random sequence generation except for three studies. As for selection bias, two studies were evaluated as unclear risk of bias for no more details in the methods of allocation, and the others as low risk of bias. Blinding of participants and personnel were performed in all but two studies. All the studies were regarded as low risk in attrition bias and reporting bias. There was no obvious other bias in all studies.

### Meta analysis

#### Anthropometric indices

##### BW

**Fig. 3:**
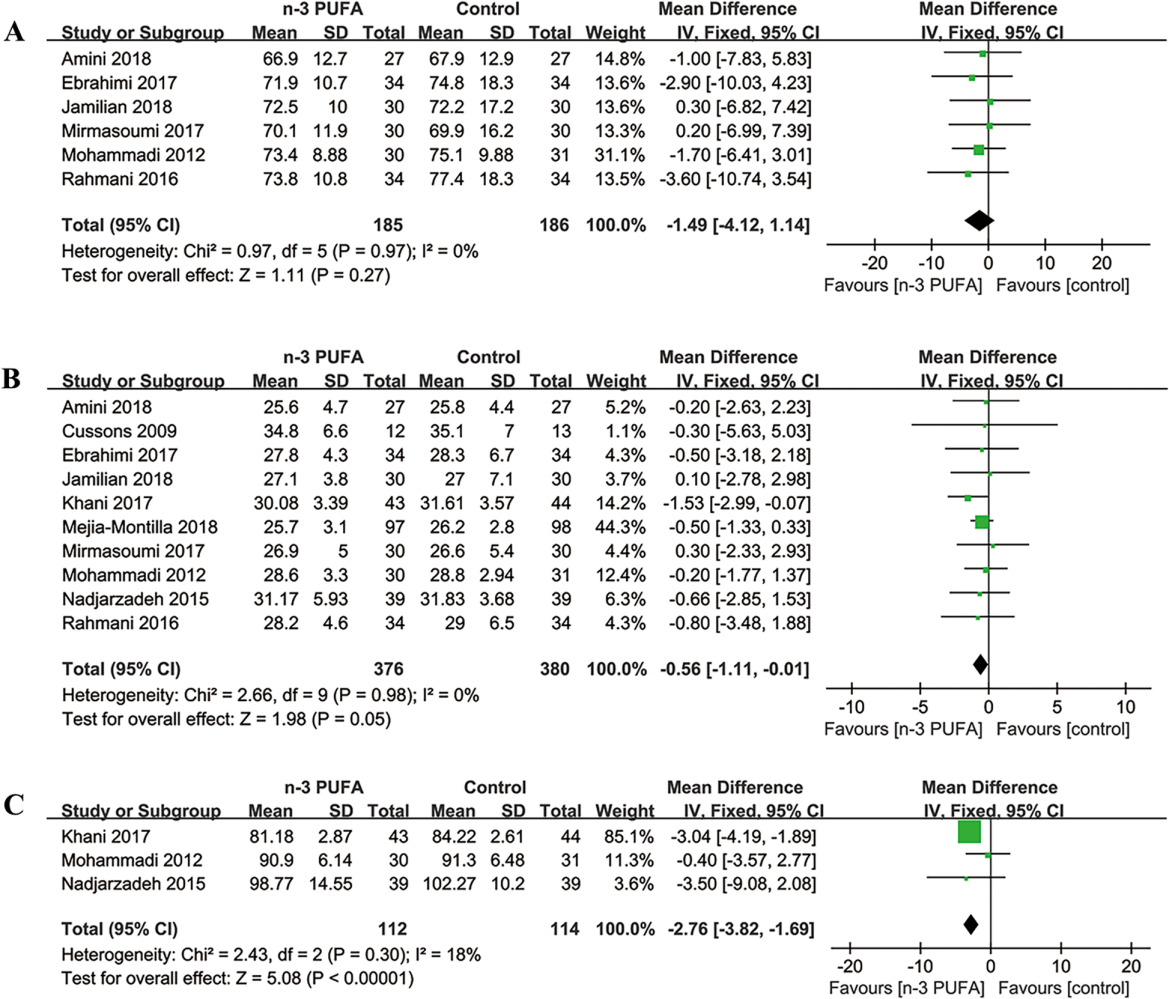
Forest plots of BW (**A**), BMI (**B**), WC (**C**) levels among PCOS patients

Six [20, 21, 29–31, 33] of the included studies (371 participants) evaluated the effects of n-3 PUFA on BW (kg). The meta-analysis showed no significant difference in BW levels (MD = -1.49; 95% CI: -4.12 to 1.14; *p* = 0.27) between the two groups with low heterogeneity (I^2^ = 0%) (Fig. 3A). The subgroup analyses showed no significant differences stratified by study duration, the source and the dosage of n-3 PUFA (Table 2).

**Table 2:** The effects of n-3 PUFA on insulin resistance indices and lipid profiles based on subgroup analysis

	Subgrouped by	No. of trials	No. of participants	Meta-analysis			Heterogeneity	
				MD	95% CI	*p*-Value	I^2^ (%)	Cochrane Q-test (*p*-Value)
BW	Study duration							
	≤ 8 weeks	1	61	-1.70	-6.41, 3.01	0.48	NA	NA
	> 8 weeks	5	310	-1.40	-4.56, 1.77	0.39	0	0.92
	Sources							
	Marine derived	3	175	-1.07	-4.47, 2.34	0.54	0	0.90
	Plant origins	3	196	-2.11	-6.24, 2.02	0.32	0	0.74
	Dosage							
	≤ 1000 mg	2	136	-3.25	-8.29, 1.80	0.21	0	0.89
	> 1000 mg	4	235	-0.84	-3.91, 2.24	0.59	0	0.96
BMI	Study duration							
	≤ 8 weeks	2	86	-0.21	-1.71, 1.30	0.79	0	0.97
	> 8 weeks	7	670	-0.61	-1.21, -0.02	0.04	0	0.93
	Sources							
	Marine derived	7	460	-0.59	-1.18, -0.00	0.05	0	0.90
	Plant origins	3	196	-0.33	-1.86, 1.21	0.68	0	0.84
	Dosage							
	≤ 1000 mg	4	409	-0.54	-1.26, 0.18	0.14	0	1.0
	> 1000 mg	6	347	-0.59	-1.45, 0.28	0.18	0	0.76
WC	Study duration							
	≤ 8 weeks	1	61	-0.40	-3.57, 2.77	0.80	NA	NA
	> 8 weeks	2	165	-3.06	-4.19, -1.93	0.00	0	0.87
	Sources							
	Marine derived	NA	NA	NA	NA	NA	NA	NA
	Plant origins	NA	NA	NA	NA	NA	NA	NA
	Dosage							
	≤ 1000 mg	1	78	-3.50	-9.08, 2.08	0.22	NA	NA
	> 1000 mg	2	148	-2.73	-3.81, -1.65	0.00	58	0.12
FPG	Study duration							
	≤ 8 weeks	2	86	-5.79	-10.18, -1.40	0.01	43	0.19
	> 8 weeks	4	269	-3.54	-5.49, -1.59	0.00	17	0.30
	Sources							
	Marine derived	4	227	-3.39	-5.47, -1.31	0.00	8	0.35
	Plant origins	2	128	-5.37	-8.84, -1.90	0.00	51	0.15
	Dosage							
	≤ 1000 mg	1	68	-7.10	-11.31, -2.89	0.00	NA	NA
	> 1000 mg	5	287	-3.22	-5.18, -1.25	0.00	0	0.47
FINS	Study duration							
	≤ 8 weeks	2	86	-1.29	-2.77, 0.20	0.09	0	0.95
	> 8 weeks	4	377	-2.84	-3.69, -1.98	0.00	0	0.85
	Sources							
	Marine derived	4	335	-2.40	-3.20, -1.61	0.00	22	0.28
	Plant origins	2	128	-2.75	-4.73, -0.77	0.00	0	0.96
	Dosage							
	≤ 1000 mg	2	263	-3.05	-4.06, -2.03	0.00	0	0.79
	> 1000 mg	4	200	-1.77	-2.86, -0.68	0.00	0	0.79
HOMA-IR	Study duration							
	≤ 8 weeks	2	86	0.82	-2.19, 3.83	0.59	91	0.00
	> 8 weeks	4	377	-0.47	-0.66, -0.29	0.00	0	0.63
	Sources							
	Marine derived	4	335	-0.30	-0.78, 0.17	0.21	74	0.00
	Plant origins	2	128	-0.75	-1.23, -0.27	0.00	0	0.84
	Dosage							
	≤ 1000 mg	2	263	-0.43	-0.65, -0.22	0.00	0	0.41
	> 1000 mg	4	200	-0.29	-0.96, 0.37	0.39	76	0.00
QUICKI	Study duration							
	≤ 8 weeks	NA	NA	NA	NA	NA	NA	NA
	> 8 weeks	NA	NA	NA	NA	NA	NA	NA
	Sources							
	Marine derived	1	54	0.01	0.00, 0.02	0.02	NA	NA
	Plant origins	2	128	0.01	0.01, 0.02	0.00	50	0.16
	Dosage							
	≤ 1000 mg	1	68	0.01	0.00, 0.02	0.04	NA	NA
	> 1000 mg	2	114	0.01	0.01, 0.02	0.00	55	0.14
Adiponectin	Study duration							
	≤ 8 weeks	1	61	1.50	0.11, 2.89	0.03	NA	NA
	> 8 weeks	2	273	1.46	1.11, 1.81	0.00	0	0.44
	Sources							
	Marine derived	NA	NA	NA	NA	NA	NA	NA
	Plant origins	NA	NA	NA	NA	NA	NA	NA
	Dosage							
	≤ 1000 mg	2	273	1.46	1.11, 1.81	0.00	0	0.44
	> 1000 mg	1	61	1.50	0.11, 2.89	0.03	NA	NA
TG	Study duration							
	≤ 8 weeks	2	86	-5.55	-16.85, 5.74	0.33	21	0.26
	> 8 weeks	5	464	-9.37	-10.61, -8.14	0.00	55	0.06
	Sources							
	Marine derived	5	422	-9.28	-10.51, -8.05	0.00	55	0.07
	Plant origins	2	128	-24.94	-48.00, -1.87	0.03	0	0.78
	Dosage							
	≤ 1000 mg	2	263	-16.71	-22.50, -10.93	0.00	0	0.47
	> 1000 mg	5	287	-8.98	-10.24, -7.72	0.00	0	0.47
TC	Study duration							
	≤ 8 weeks	2	86	-13.42	-26.67, -0.17	0.05	0	0.36
	> 8 weeks	5	464	-12.20	-20.18, -4.22	0.00	79	0.00
	Sources							
	Marine derived	5	422	-12.18	-20.45, -3.92	0.00	79	0.00
	Plant origins	2	128	-12.91	-24.77, -1.05	0.03	0	0.33
	Dosage							
	≤ 1000 mg	2	263	-20.97	-26.04, -15.90	0.00	0	0.58
	> 1000 mg	5	287	-9.08	-11.55, -6.60	0.00	0	0.65
HDL-C	Study duration							
	≤ 8 weeks	2	86	0.64	-2.06, 3.35	0.64	0	0.78
	> 8 weeks	5	464	2.60	0.07, 5.14	0.04	81	0.00
	Sources							
	Marine derived	5	422	3.09	0.65, 5.52	0.01	81	0.00
	Plant origins	2	128	-0.65	-4.08, 2.78	0.71	0	0.76
	Dosage							
	≤ 1000 mg	2	263	2.27	-3.57, 8.10	0.45	83	0.01
	> 1000 mg	5	287	1.52	-2.30, 5.35	0.43	83	0.00
LDL-C	Study duration							
	≤ 8 weeks	2	86	-9.98	-25.36, 5.40	0.20	22	0.26
	> 8 weeks	5	464	-10.86	-21.05, -0.68	0.04	94	0.00
	Sources							
	Marine derived	5	422	-11.87	-22.46, -1.28	0.03	94	0.00
	Plant origins	2	128	-6.97	-17.79, 3.86	0.21	0	0.39
	Dosage							
	≤ 1000 mg	2	263	-19.45	-34.19, -4.71	0.01	79	0.03
	> 1000 mg	5	287	-9.50	-10.74, -8.27	0.00	1	0.40
VLDL-C	Study duration							
	≤ 8 weeks	NA	NA	NA	NA	NA	NA	NA
	> 8 weeks	NA	NA	NA	NA	NA	NA	NA
	Sources							
	Marine derived	1	54	1.70	-3.92, 7.32	0.55	NA	NA
	Plant origins	2	128	-5.05	-9.66, -0.44	0.03	0	0.78
	Dosage							
	≤ 1000 mg	1	68	-5.60	-11.68, 0.48	0.07	NA	NA
	> 1000 mg	2	114	-0.62	-5.02, 3.78	0.78	41	0.19
hs-CRP	Study duration							
	≤ 8 weeks	2	86	0.03	-0.37, 0.43	0.89	0	0.92
	> 8 weeks	4	234	-0.83	-1.69, 0.04	0.06	71	0.02
	Sources							
	Marine derived	4	422	-11.87	-22.46, -1.28	0.03	94	0.00
	Plant origins	2	128	-6.97	-17.79, 3.86	0.21	0	0.39
	Dosage							
	≤ 1000 mg	1	60	0.20	-0.47, 0.87	0.56	NA	NA
	> 1000 mg	5	260	-0.82	-1.62, -0.02	0.04	68	0.01

*Abbreviations: BW* body weight, *BMI* body mass index, *FPG* fasting plasma glucose, *FINS* fasting insulin, *HDL-C* high-density lipoprotein cholesterol, *HOMA-IR* homeostatic model of assessment for insulin resistance, *hs-CRP* high sensitivity C-reactive protein, *LDL-C* low-density lipoprotein cholesterol, *QUICK* quantitative insulin sensitivity check index, *TC* total cholesterol, *TG* triglycerides, *VLDL-C* very low density lipoprotein-cholesterol, *MD* mean difference, *NA* not applicable

##### BMI

Ten [19–21, 26, 29–34] of the included studies (756 participants) evaluated the effects of n-3 PUFA on BMI (kg/m^2^). The meta-analysis showed no significant difference in BMI levels (MD = -0.56; 95% CI: -1.11 to -0.01; *p* = 0.05) between the two groups with low heterogeneity (I^2^ = 0%) (Fig. 3B). The subgroup analyses showed no significant differences stratified by the source and the dosage of n-3 PUFA, but lower BMI in n-3 PUFA group compared with control group in duration > 8 weeks (Table 2).

##### WC

Three [19, 31, 34] of the included studies (226 participants) evaluated the effects of n-3 PUFA on WC (cm) levels. The meta-analysis showed WC levels lowered significantly in n-3 PUFA group (MD = -2.76, 95% CI: -3.82 to -1.69; *p* < 0.00001) (Fig. 3C). There was low heterogeneity between the two groups (I^2^ = 18%). The subgroup analyses showed significantly lower WC levels in n-3 PUFA group than those in control group with study duration > 8 weeks and dosage > 1000 mg/d (Table 2).

#### The metabolic status in IR

##### FPG

**Fig. 4:**
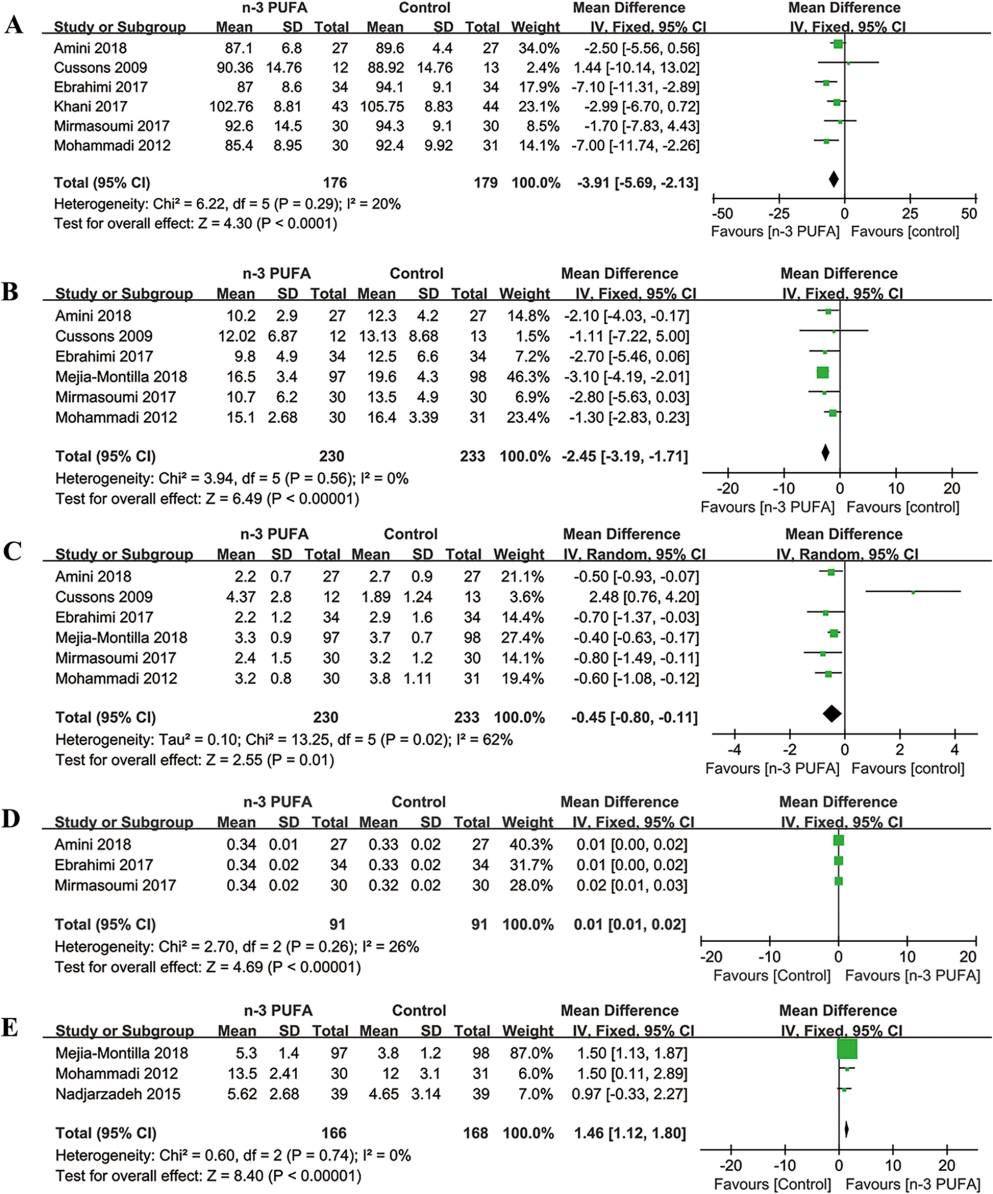
Forest plots of FPG (**A**), FINS (**B**), HOMA-IR (**C**), QUICKI (**D**) and Adiponectin (**E**) levels among PCOS patients

Six [19–21, 30–32] of the included studies (355 participants) evaluated the effects of n-3 PUFA on FPG (mg/dL) levels. The meta-analysis showed FPG levels lowered significantly in n-3 PUFA group compared with control group (MD = -3.91, 95% CI: -5.69 to -2.13; *p* < 0.0001) (Fig. 4A). There was low heterogeneity between the two groups (I^2^ = 20%). The subgroup analyses showed no significant differences stratified by study duration, the source and the dosage of n-3 PUFA (Table 2).

##### FINS

Six [19–21, 26, 29–32] of the included studies (463 participants) evaluated the effects of n-3 PUFA on FINS (µIU/mL) levels. Compared with control group, lower FINS levels were in n-3 PUFA group (MD = -2.45, 95% CI: -3.19 to -1.71; *p* < 0.00001) with low heterogeneity (I^2^ = 0%) (Fig. 4B). The subgroup analyses showed no significant differences stratified by the source and the dosage of n-3 PUFA, but lower FINS levels in n-3 PUFA group than in control group with the study duration > 8 weeks (Table 2).

##### HOMA-IR

Six [19–21, 26, 29–32] of the included studies (463 participants) reported the effects of n-3 PUFA on HOMA-IR. The meta-analysis showed a reduction in HOMA-IR (MD = -0.45, 95% CI: -0.80 to -0.11; *p* = 0.01) in n-3 PUFA group compared with control group with moderate heterogeneity (I^2^ = 62%) (Fig. 4C). The subgroup analyses showed significantly lower HOMA-IR levels in n-3 PUFA group than in control group with study duration > 8 weeks, plant origin and dosage ≤ 1000 mg/d (Table 2).

##### QUICKI

Three [20, 21, 30] of the included studies (182 participants) evaluated the effects of n-3 PUFA on QUICKI. The meta-analysis showed QUICKI was increased significantly in n-3 PUFA group compared with control group (MD = 0.01, 95% CI: 0.01 to 0.02; *p* < 0.00001) (Fig. 4D). There was low heterogeneity between the two groups (I^2^ = 26%). The subgroup analyses showed no significant differences stratified by the source and the dosage of n-3 PUFA (Table 2).

##### Adiponectin

Three [26, 31, 34] of the included studies (334 participants) evaluated the effects of n-3 PUFA on Adiponectin (ng/mL) levels. The meta-analysis showed Adiponectin levels increased significantly in n-3 PUFA group compared with control group (MD = 1.46, 95% CI: 1.12 to 1.80; *p* < 0.00001) (Fig. 4E). There was low heterogeneity between the two groups (I^2^ = 0%). However, the subgroup analyses showed no significant difference stratified by study duration and the dosage of n-3 PUFA (Table 2).

#### The metabolic status in lipid profiles

**Fig. 5:**
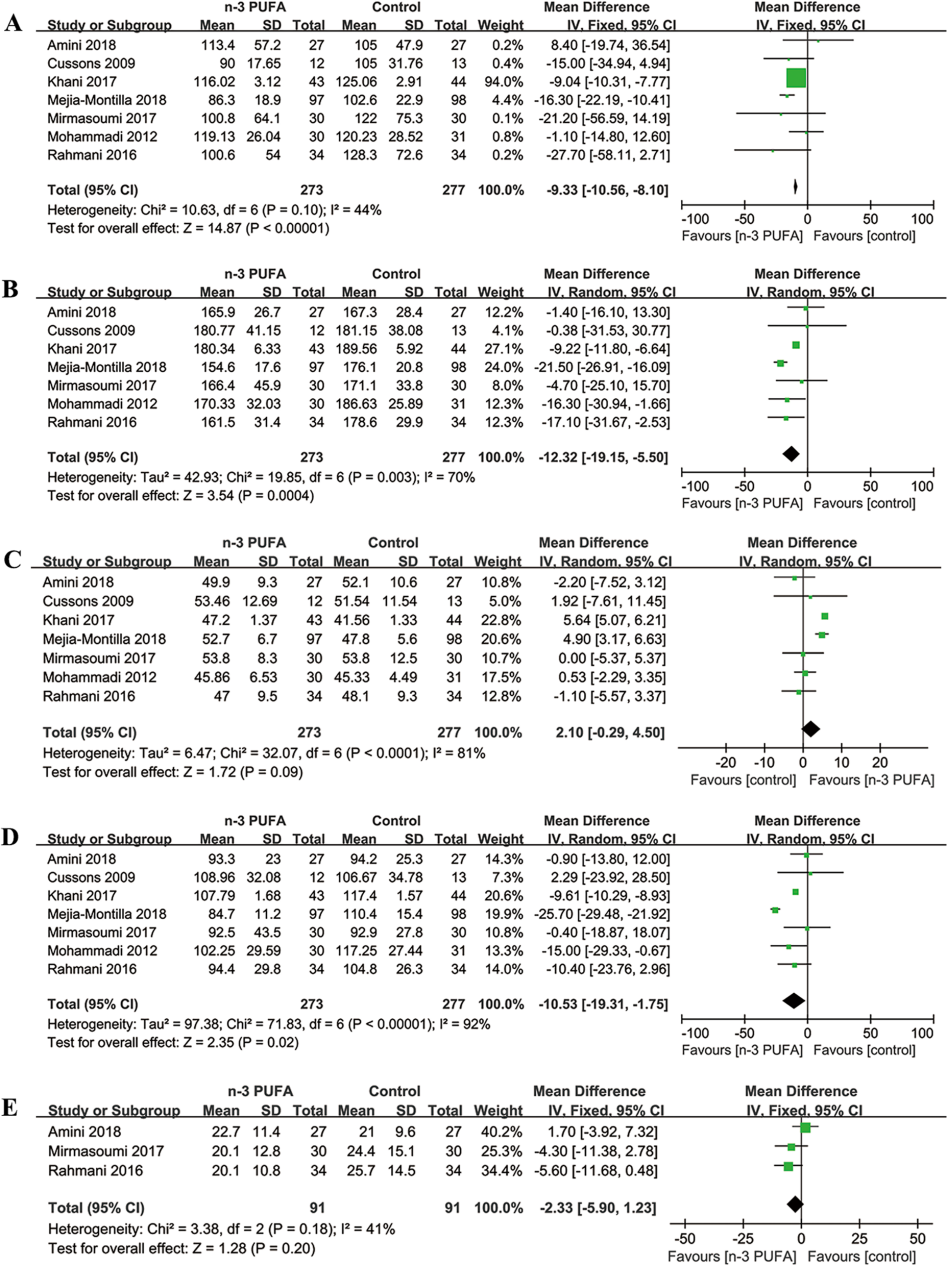
Forest plots of TG (**A**), TC (**B**), HDL-C (**C**), LDL-C (**D**) and VLDL-C (**E**) levels among PCOS patients

***TG.*** Seven [19–21, 26, 29, 31, 32] of the included studies (550 participants) evaluated the effects of n-3 PUFA on TG (mg/dL) levels. The meta-analysis showed a reduction in TG levels (MD = -9.33, 95% CI: -10.56 to -8.10; *p* < 0.00001) in n-3 PUFA group with low heterogeneity (I^2^ = 44%) (Fig. 5A). The subgroup analysis showed no significant differences stratified by the source and the dosage of n-3 PUFA, but lower TG levels in n-3 PUFA group than in control group with study duration > 8 weeks (Table 2).

##### TC

Seven [19–21, 26, 29, 31, 32] of the included studies (550 participants) evaluated the effects of n-3 PUFA on TC (mg/dL) levels. The meta-analysis showed TC levels were significantly lower in n-3 PUFA group (MD = -12.32, 95% CI: -19.15 to -5.50; *p* = 0.0004) compared with control group with moderate heterogeneity (I^2^ = 70%) (Fig. 5B). The subgroup analysis showed no significant differences stratified by the source and the dosage of n-3 PUFA, but lower TC levels in n-3 PUFA group than in control group with study duration > 8 weeks (Table 2).

##### HDL-C

Seven [19–21, 26, 29, 31, 32] of the included studies (550 participants) evaluated the effects of n-3 PUFA on HDL-C (mg/dL) levels. The meta-analysis showed no significant difference in HDL-C levels (MD = 2.10; 95% CI: -0.29 to 4.5; *p* = 0.09) between the two groups with significant heterogeneity (I^2^ = 81%) (Fig. 5C). The subgroup analysis showed no significant difference stratified by the dosage of n-3 PUFA, but significant increases of HDL-C levels in n-3 PUFA group than in control group with study duration > 8 weeks and marine derived (Table 2).

##### LDL-C

Seven [19–21, 26, 29, 31, 32] of the included studies (550 participants) evaluated the effects of n-3 PUFA on LDL-C (mg/dL) levels. The meta-analysis showed LDL-C levels were lower in n-3 PUFA group (MD = -10.53, 95% CI: -19.31 to -1.75; *p* = 0.02) compared with control group with significant heterogeneity (I^2^ = 92%) (Fig. 5D). The subgroup analysis showed no significant difference stratified by the dosage of n-3 PUFA, but significant decreases of LDL-C levels in n-3 PUFA group than in control group with study duration > 8 weeks and marine derived (Table 2).

##### VLDL-C

Three [20, 21, 29] of the included studies (182 participants) evaluated the effects of n-3 PUFA on VLDL-C (mg/dL) levels. The meta-analysis showed no significant difference in VLDL-C levels (MD = -2.33; 95% CI: -5.90 to 1.23; *p* = 0.20) between the two groups with low heterogeneity (I^2^ = 41%) (Fig. 5E). The subgroup analysis showed no significant difference stratified by the dosage of n-3 PUFA, but a significant decrease of VLDL-C levels in n-3 PUFA group than in control group with plant origin (Table 2).

##### hs-CRP

**Fig. 6:**
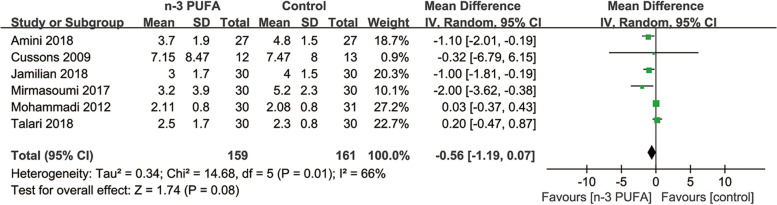
Forest plots of hs-CRP levels among PCOS patients

Six [5, 10, 18, 20, 25, 30] of the included studies (320 participants) evaluated the effects of n-3 PUFA on hs-CRP (mg/L) levels. The meta-analysis showed no significant difference in hs-CRP (MD = -0.56; 95% CI: -1.19 to 0.07; *p* = 0.08) between the two groups with moderate heterogeneity (I^2^ = 66%) (Fig. 6). The subgroup analysis showed no significant difference stratified by the study duration of n-3 PUFA, but significant decreases of hs-CRP levels in n-3 PUFA group than in control group with marine derived and study duration > 1000 mg/d (Table 2).

### Sensitivity analysis and publication bias

**Fig. 7:**
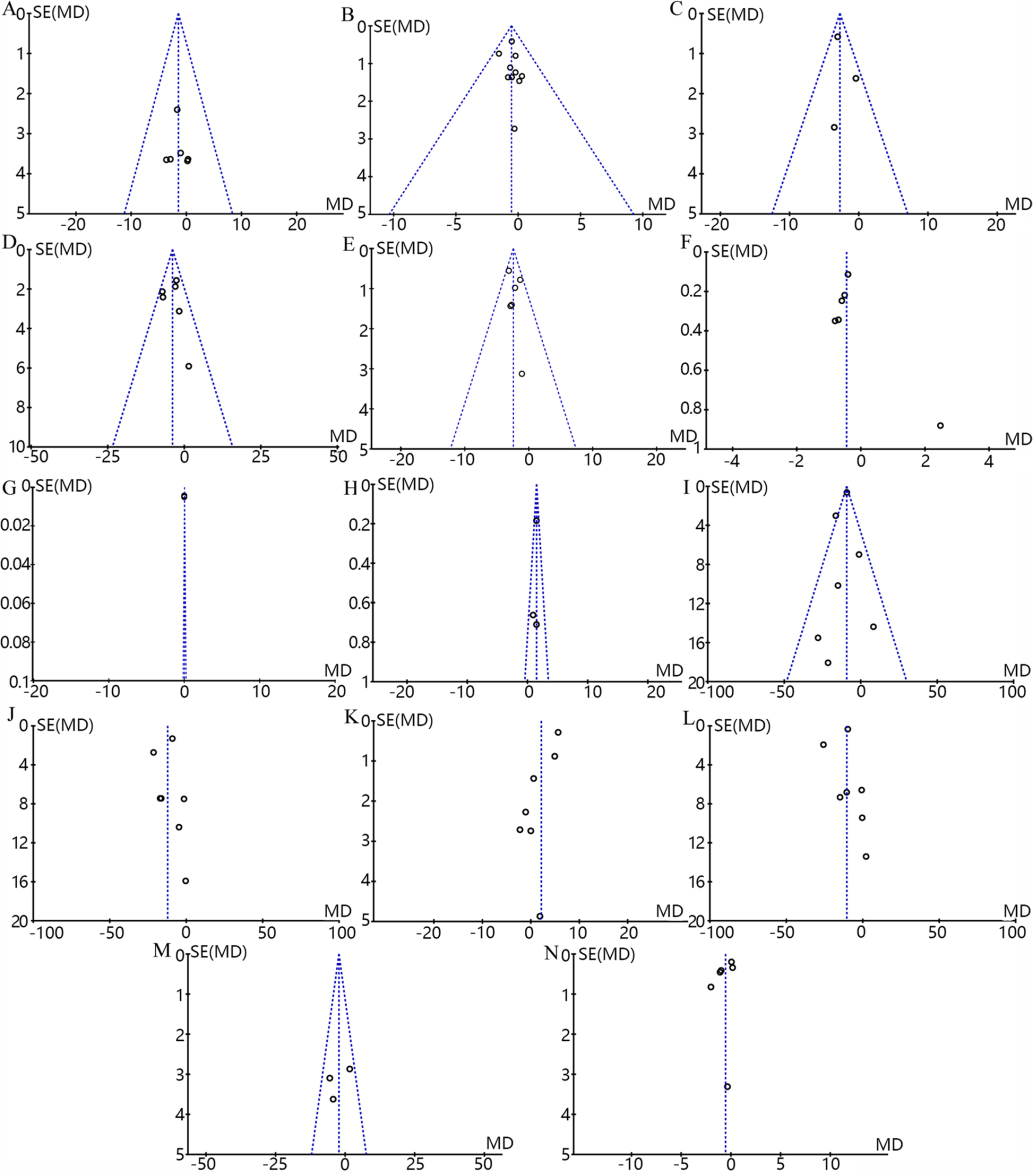
Funnel plots of BW (**A**), BMI (**B**), WC (**C**), FPG (**D**), FINS (**E**), HOMA-IR (**F**), QUICKI (**G**), Adiponectin (**H**), TG (**I**), TC (**J**), HDL-C (**K**), LDL-C (**L**), VLDL-C (**M**) and hs-CRP (**N**) levels among PCOS patients

The results of sensitivity analysis show that most of the conclusions are stable. But, heterogeneity of HDL-C (I^2^ = 81%) was decreased (I^2^ = 68%) after rejecting one study conducted by Khani et al. [19], the result (MD = -1.10; 95% CI: -5.57 to 3.37; *p* = 0.10) resembled the former (Fig. S1a). After rejecting the study of Mejia-Montilla et al. [26], heterogeneity of LDL-C (I^2^ = 92%) was completely eliminated (I^2^ = 0%), and there was a significant decrease in LDL-C levels after replenishing n-3 PUFA (MD = -10.40; 95% CI: -23.76 to 2.96; *p* < 0000.1) (Fig. S1b). We assessed publication bias by funnel plot and found that the shapes of funnel plots of BW, BMI, WC, FPG, FINS, TG, and VLDL-C in our meta-analysis were relatively symmetric, indicating no publication bias (Fig. 7A-E, I, M). But, some publication bias may exist in remaining indicators for asymmetrical funnel plots (Fig. 7F-H, J-L, N), which may also be related to the limited included studies.

### Certainty assessment

GRADE approach was used to assess the quality of evidence for each comparison (Table 3). For BMI, FINS and TG, the quality of evidence was considered to be high, while for these six indicators (WC, FPG, HOMA-IR, TC and LDL-C), the quality of evidence was moderate. For BW, QUICK, Adiponectin, HDL-C, and VLDL-C, GRADE evaluation results were low quality, and the evaluation for hs-CRP was very low quality.

**Table 3:** Quality of evidence according to GRADE approach

	**Certainty assessment**							**№ of patients**		**Effect**		**Certainty**	**Importance**
	**№ of studies**	**Study design**	**Risk of bias**	**Inconsistency**	**Indirectness**	**Imprecision**	**Other considerations**	**n-3 PUFA**	**control**	**Relative** **(95% CI)**	**Absolute** **(95% CI)**		
**BW**													
	6	randomised trials	not serious	not serious	not serious	very serious^a,b^	none	185	186	-	MD **1.49 lower** (4.12 lower to 1.14 higher)	⨁⨁◯◯ Low	NOT IMPORTANT
**BMI**													
	10	randomised trials	not serious	not serious	not serious	not serious	none	376	380	-	MD **0.56 lower** (1.11 lower to 0.01 lower)	⨁⨁⨁⨁ High	NOT IMPORTANT
**WC**													
	3	randomised trials	not serious	not serious	not serious	serious^a^	none	112	114	-	MD **2.76 lower** (3.82 lower to 1.69 lower)	⨁⨁⨁◯ Moderate	NOT IMPORTANT
**FPG**													
	6	randomised trials	not serious	not serious	not serious	serious^a^	none	176	179	-	MD **3.91 lower** (5.69 lower to 2.13 lower)	⨁⨁⨁◯ Moderate	IMPORTANT
**FINS**													
	6	randomised trials	not serious	not serious	not serious	not serious	none	230	233	-	MD **2.45 lower** (3.19 lower to 1.71 lower)	⨁⨁⨁⨁ High	IMPORTANT
**HOMA-IR**													
	6	randomised trials	serious^c^	not serious	not serious	not serious	publication bias strongly suspected^c^	230	233	-	MD **0.45 lower** (0.8 lower to 0.11 lower)	⨁⨁◯◯ Low	IMPORTANT
**QUICKI**													
	3	randomised trials	serious^c^	not serious	not serious	serious^a^	publication bias strongly suspected^c^	91	91	-	MD **0.01 higher** (0.01 higher to 0.02 higher)	⨁◯◯◯ Very low	IMPORTANT
**Adiponectin**													
	3	randomised trials	serious^c^	not serious	not serious	serious^a^	publication bias strongly suspected^c^	166	168	-	MD **1.46 higher** (1.12 higher to 1.8 higher)	⨁◯◯◯ Very low	IMPORTANT
**TG**													
	7	randomised trials	not serious	not serious	not serious	not serious	none	273	277	-	MD **9.33 lower** (10.56 lower to 8.1 lower)	⨁⨁⨁⨁ High	IMPORTANT
**TC**													
	7	randomised trials	serious^c^	not serious	not serious	not serious	publication bias strongly suspected^c^	273	277	-	MD **12.32 lower** (19.15 lower to 5.5 lower)	⨁⨁◯◯ Low	IMPORTANT
**HDL-C**													
	7	randomised trials	serious^c^	serious^d^	not serious	serious^b^	publication bias strongly suspected^c^	273	277	-	MD **2.1 higher** (0.29 lower to 4.5 higher)	⨁◯◯◯ Very low	IMPORTANT
**LDL-C**													
	7	randomised trials	serious^c^	not serious	not serious	not serious	publication bias strongly suspected^c^	273	277	-	MD **10.53 lower** (19.31 lower to 1.75 lower)	⨁⨁◯◯ Low	IMPORTANT
**VLDL-C**													
	3	randomised trials	not serious	not serious	not serious	very serious^a,b^	none	91	91	-	MD **2.33 lower** (5.9 lower to 1.23 higher)	⨁⨁◯◯ Low	IMPORTANT
**hs-CRP**													
	6	randomised trials	serious^c^	not serious	not serious	very serious^a,b^	publication bias strongly suspected^c^	159	161	-	MD **0.56 lower** (1.19 lower to 0.07 higher)	⨁◯◯◯ Very low	IMPORTANT

*Abbreviations: CI* confidence interval, *MD* mean difference, *BW* body weight, *BMI* body mass index, *FPG* fasting plasma glucose, *FINS* fasting insulin, *HDL-C* high-density lipoprotein cholesterol, *HOMA-IR* homeostatic model of assessment for insulin resistance, *hs-CRP* high sensitivity C-reactive protein, *LDL-C* low-density lipoprotein cholesterol, *QUICK* quantitative insulin sensitivity check index, *TC* total cholesterol, *TG* triglycerides, *VLDL-C* very low density lipoprotein-cholesterol

^a^Total sample size is less than optimal information size

^b^Effect size confidence intervals were too large

^c^Publication bias may exist; d. There is methodological heterogeneity

## Discussion

This meta-analysis summarizes the effects of n-3 PUFA on metabolic status (insulin metabolism and lipid metabolism) in patients with PCOS, which deepens our understanding of the role of n-3 PUFA in women with PCOS. We found that n-3 PUFA ameliorated metabolic status of PCOS women, reducing FPG, FINS, HOMA-IR, QUICKI, Adiponectin, TG, TC and LDL-C, but didn’t modulate HDL-C, VLDL-C and hs-CRP levels. According to study duration, the source and dosage of n-3 PUFA, we conducted subgroup analyses and found that n-3 PUFA ameliorated FPG and Adiponectin in patients with treatment duration ≤ 8 weeks, ameliorated BMI, WC, FPG, FINS, HOMA-IR, Adiponectin, TG, TC, HDL-C and LDL-C in patients with treatment duration > 8 weeks, reduced FPG, FINS, QUICKI, TG, TC, HDL-C, LDL-C and hs-CRP in n-3 PUFA group with marine derived, and decreased FPG, FINS, HOMA-IR, QUICKI, TG, TC and VLDL-C in n-3 PUFA group with plant origins, ameliorated FPG, FINS, HOMA-IR, QUICKI, Adiponectin, TG, TC and LDL-C in n-3 PUFA group with dosage ≤ 1000 mg/d, and decreased WC, FPG, FINS, QUICKI, Adiponectin, TG, TC and LDL-C and hs-CRP in n-3 PUFA group with dosage > 1000 mg/d.

PCOS women are vulnerable to abnormal insulin metabolism [20]. The prevalence of PCOS accompanied with IR ranged from 44 to 70% [4]. IR and hyperinsulinemia are considered to be the important pathological and physiological basis of PCOS [36]. Insulin plays an important role in regulating energy metabolism and growth of human body. In the ovary, insulin is closely related to ovulation and egg quality. Our results demonstrated that n-3 PUFA lower FPG, FINS and HOMA-IR levels, which was also supported by subgroup analyses, indicating the robustness and reliability of our results. N-3 PUFA might reduce FPG level by enhancing the sensitivity of insulin signal induced by G protein-coupling receptors of glucagon like peptide 1 (GLP-1), and regulating the signal pathways of insulin production [37]. In addition, Adiponectin (an insulin-sensitizing hormone) levels increased significantly after n-3 PUFA supplements, which also shows that n-3 PUFA improves IR in PCOS. Our findings were consistent with Oner’s clinical study [22]. However, a previous meta-analysis [22] included three RCTs involving 72 cases and 73 controls indicated that supplementation of n-3 PUFA might not relieve IR in women with PCOS. The results inconsistent with ours may be related to the smaller number of included trials and samples size in those studies.

The mechanism of n-3 PUFA improving insulin sensitivity might be interpreted by the inhibition of nuclear factor-κB (NF-κB) transcription factor and pro-inflammatory mediators, thereby reducing IR [38]. Thus, hs-CRP, which is a very sensitive inflammatory marker in the blood, has been decreased, especially in n-3 PUFA group with marine derived, study duration > 8 weeks and dosage > 1000 mg/d. In addition to IR, PCOS usually increases the risk of various cardiac metabolic abnormalities [3]. The overall meta-analyses demonstrated that n-3 PUFA reduced TC level, but didn’t modify TG, HDL-C, LDL-C levels with high heterogeneity. The subgroup analysis based on dosage showed significant reduction on the heterogeneity of overall result of TG, indicating that different doses of n-3 PUFA had varying influences on the TG in PCOS patients, and the possibility of dose dependence could not be excluded. The high heterogeneity of HDL-C was also eliminated after sensitivity analyses by removing one study conducted by Khani et al. [19], which used NIH diagnostic criteria, while Rotterdam 2003 criteria were utilized in other studies. Compared to NIH diagnostic criteria, Rotterdam 2003 criteria is more applicable to clinical practice, which defines four PCOS phenotypes, so that patients with relatively "mild" symptoms or without sparse ovulation or high androgen presentation could be included in the diagnosis. The results of subgroup analyses showed that n-3 PUFA with marine derived and study duration > 8 weeks evidently lowered HDL-C levels, which are inconsistent with the results of HDL-C in overall meta-analysis. This may suggest that it was the n-3 PUFA with marine derived and study duration > 8 weeks that had a moderating effect on HDL-C.

A clinical study was done among 60 women with PCOS treated with 1,000 mg of n-3 PUFA daily for 12 weeks, finding that n-3 PUFA significantly down-regulated the expression of oxidized low density lipoprotein receptor (LDL-R) in peripheral blood of PCOS patients. And n-3 PUFA might increase TG lipolysis and reduce TG uptake in liver circulation through enhancing lipoprotein lipase activity [39]. Adenosine 5’-monophosphate (AMP)-activated protein kinase (AMPK) pathway was the main sensor of cell energy status, which regulates the distribution between lipid oxidation and lipid metabolism. The previous study showed that the effect of n-3 PUFA on lipid distribution was partly mediated by enhancing AMPK pathway [40].

It is worth mentioning that compared with the existing drugs for the treatment of PCOS in the clinic, n-PUFA has its own advantages and disadvantages. Metformin is often used to improve IR in PCOS, but it has side effects such as diarrhea and gastrointestinal discomfort [41]. So far, n-3 PUFA has been reported to have no side effects. In addition, n-3 PUFA can lower blood lipids in addition to improving IR. But the cost of n-PUFA is higher than metformin. However, dietary therapy also maybe a good option for some patients who cannot tolerate drug therapy.

This meta-analysis has several strengths. Compared with previous systematic studies [22, 42, 43], our study included the largest number of RCTs and covered more comprehensive indicators on anthropometric indices, lipid profiles, IR and inflammatory parameters, which are important for a comprehensive understanding of the metabolic status of PCOS patients. In addition, we performed subgroup analyses based on study duration, sources and dosage of n-3 PUFA. Subgroup analysis can help to understand the source of heterogeneity and also provide better decision making for a certain type of patients. Our study confirm that longer courses (> 8 weeks) of n-3 PUFA treatment improved more metrics about IR and lipid profiles, including BMI, WC, FPG, FINS, HOMA-IR, Adiponectin, TG, TC, HDL-C and LDL-C. But, different sources and dosages of n-3 PUFA seemed to have similar effect on some indicators, like FPG, FINS, QUICKI, TG, TC, but have differentiated influence on certain metrics, such as HDL-C, LDL-C and hs-CRP. We can select the appropriate types and economical dosage of n-3 PUFA according to the patient’s basic condition. What’s more, we report the results based on the GRADE approach, which provides a systematic approach for making clinical practice recommendations.

However, these limitations should not be ignored in our paper. Firstly, the number of studies investigating the effect of n-3 PUFA on metabolic status in women with PCOS was still less. Secondly, the number of patients in the enrolled studies was small, which inevitably affected the credibility of the results and increased heterogeneity. Thirdly, some studies used varying doses (400 IU/d-50000 IU/2 weeks) of vitamin E, which may be a source of heterogeneity. A recent published meta-analysis [44] has demonstrated that vitamin E supplementation improves lipid profile, decreases insulin and HOMA-IR levels in PCOS. At last, most of the studies were conducted in Iran, we cannot guarantee that the findings of this review can be applied to countries outside of Iran. Thus, more RCTs with large samples conducted in other countries are needed to extend the therapeutic effectiveness.

## Conclusions

This meta-analysis indicated that n-3 PUFA could ameliorated metabolic status of women with PCOS, by reducing FPG, FINS, HOMA-IR, QUICKI, Adiponectin, TG, TC and LDL-C levels, but couldn’t affect HDL-C, VLDL-C and hs-CRP levels. According to results of subgroup analyses based on study duration, the source and dosage of n-3 PUFA, n-3 PUFA with study duration > 8 weeks is more conducive to improve the metabolic status in insulin resistance and lipid profiles. The meta-analysis demonstrates that n-3 PUFA may be an effective intervention for alleviating metabolic status in PCOS. Hence, we recommend PCOS patients replenish n-3 PUFA with duration > 8 weeks regardless of the source and the dosage to retard the pathogenesis of PCOS related metabolic diseases. More large scale RCTs are needed to confirm the idea.

## Supplementary Information

**Additional file 1:** **Supplementary Tables.**

**Additional file 2:** **Supplementary Figure.**

## Data Availability

All data is available from the corresponding author on reasonable request.

## Abbreviations

AMPK: Adenosine 5’-monophosphate (AMP)-activated protein kinase
BW: Body weight
BMI: Body mass index
FINS: Fasting Insulin
FPG: Fasting plasma glucose
GLP-1: G protein receptor of glucagon like peptide 1
HDL-C: High-density lipoprotein cholesterol
HOMA-IR: Homeostatic model assessment-insulin resistance
hs-CRP: High sensitivity C-reactive protein
IR: Insulin resistance
LDL-C: Low-density lipoprotein cholesterol
LDLR: Low density lipoprotein receptor
MD: Mean difference
n-3 PUFA: N-3 polyunsaturated fatty Acid
NF-κB: Nuclear factor-κB
PCOS: Polycystic ovary syndrome
QUICKI: Quantitative insulin sensitivity check index
TC: Total cholesterol
TG: Triglycerides
VLDL: Very low density lipoprotein
WC: Waist circumference
