# Supplementary material for: Effects of n-3 polyunsaturated fatty acid on metabolic status in women with polycystic ovary syndrome: a meta-analysis of randomized controlled trials
Source: J Ovarian Res. 2023 Mar 17;16:54. doi: 10.1186/s13048-023-01130-4 (PMC10022207; doi:10.1186/s13048-023-01130-4)
Supplement: Supplementary file 1 — Additional file 1: Supplementary Tables. [file 13048_2023_1130_MOESM1_ESM.pdf]

Table S1. Search Strategy for Pubmed

Database	Search Strategy
PubMed	"Fatty Acids, Omega-3"[Mesh] OR "n 3 polyunsaturated fatty acid"[Title/Abstract] OR "omega 3 fatty acid"[Title/Abstract] OR "acid omega 3 fatty"[Title/Abstract] OR "fatty acid omega 3"[Title/Abstract] OR "omega 3 fatty acids"[Title/Abstract] OR "oil n 3"[Title/Abstract] OR "n 3 oil"[Title/Abstract] OR "oil n3"[Title/Abstract] OR "n 3 fatty acids"[Title/Abstract] OR "omega 3 fatty acids"[Title/Abstract] OR "pufa n 3"[Title/Abstract] OR "n 3 pufa"[Title/Abstract] OR "n3 fatty acid"[Title/Abstract] OR "n3 pufa"[Title/Abstract] OR "pufa n3"[Title/Abstract] OR "n3 polyunsaturated fatty acid"[Title/Abstract] OR "n 3 oils"[Title/Abstract] OR "n 3 fatty acid"[Title/Abstract] OR "acid n 3 fatty"[Title/Abstract] OR "fatty acid n 3"[Title/Abstract] OR "n 3 fatty acid"[Title/Abstract] OR omega-3 [Title/Abstract] OR “n-3 Polyunsaturated fatty acids” [Title/Abstract] OR “omega-3 polyunsaturated fatty acid” [Title/Abstract]<br>OR<br>“fish oil” [Title/Abstract] OR “fish oils” [Title/Abstract] OR promega[Title/Abstract] OR “super epa” [Title/Abstract] OR superepa[Title/Abstract] OR “tuna oil” [Title/Abstract] OR “tuna oils” [Title/Abstract] OR icosapentaenoic[Title/Abstract] OR docosahexaenoic[Title/Abstract] OR linolenic[Title/Abstract] OR eicosapentaenoic[Title/Abstract] OR alpha-linolenic[Title/Abstract] OR EPA[Title/Abstract] OR DHA[Title/Abstract]<br>AND<br>"polycystic ovary syndrome"[MeSH Terms] OR "ovary syndrome polycystic"[Title/Abstract] OR "syndrome polycystic ovary"[Title/Abstract] OR "stein leventhal syndrome"[Title/Abstract] OR "stein leventhal syndrome"[Title/Abstract] OR "syndrome stein leventhal"[Title/Abstract] OR "sclerocystic ovarian degeneration"[Title/Abstract] OR "sclerocystic ovary syndrome"[Title/Abstract] OR "polycystic ovarian syndrome"[Title/Abstract] OR "ovarian syndrome polycystic"[Title/Abstract] OR "polycystic ovary syndrome"[Title/Abstract] OR "sclerocystic ovaries"[Title/Abstract] OR "sclerocystic ovary"[Title/Abstract]

Table S2. Specific indicators and values involved in all included studies

Study	Adiponectin (ng/ml)		Apo-B(g/L)		Apo-A(g/L)		BW (kg)		BMI (kg/m2)		WC(cm)	
	Intervention	Control	Intervention	Control	Intervention	Control	Intervention	Control	Intervention	Control	Intervention	Control
Amini 2018	-	-	-	-	-	-	66.9±12.7	67.9±12.9	25.6±4.7	25.8±4.4	-	-
Cussons 2009	-	-	-	-	-	-	-	-	34.8±6.6	35.1±7.0	-	-
Ebrahimi 2017	-	-	-	-	-	-	71.9±10.7	74.8±18.3	27.8±4.3	28.3±6.7	-	-
Jamilian 2018	-	-	-	-	-	-	72.5±10.0	72.2±17.2	27.1±3.8	27.0±7.1	-	-
Khani 2017	-	-	-	-	-	-	-	-	30.08±3.39	31.61±3.57	81.18±2.87	84.22±2.61
Mejia-Montilla 2018	5.3±1.4	3.8±1.2	94.5±12.6	86.2±12.9	118.0±17.0	119.1±15.6	-	-	25.7±3.1	26.2±2.8	-	-
Mirmasoumi 2017	-	-	-	-	-	-	70.1±11.9	69.9±16.2	26.9±5.0	26.6±5.4	-	-
Mohammadi 2012	13.5±2.41	12.0±3.10	-	-	-	-	73.4±8.88	75.1±9.88	28.6±3.30	28.8±2.94	90.9±6.14	91.3±6.48
Nadjarzadeh 2015	5.62±2.68	4.65±3.14	-	-	-	-	-	-	31.17±5.93	31.83±3.68	98.77±14.55	102.27±10.20
Rahmani 2016	-	-	-	-	-	-	73.8±10.8	77.4±18.3	28.2±4.6	29.0±6.5	-	-
Talari 2018	-	-	-	-	-	-	-	-	-	-	-	-

Table S2. Continued

Study	hs-CRP (mg/L)		QUICKI		FPG (mg/dL)		FINS (μIU/mL)		HOMA-IR		TG (mg/dL)	
	Intervention	Control	Intervention	Control	Intervention	Control	Intervention	Control	Intervention	Control	Intervention	Control
Amini 2018	3.7±1.9	4.8±1.5	0.34±0.01	0.33±0.02	87.1±6.8	89.6±4.4	10.2±2.9	12.3±4.2	2.2±0.7	2.7±0.9	113.4±57.2	105±47.9

Cussons 2009	7.15±8.47	7.47±8.00	-	-	90.36±14.76	88.92±14.76	12.02±6.87	13.13±8.68	4.37±2.8	1.89±1.24	90±17.65	105±31.76
Ebrahimi 2017	-	-	0.34±0.02	0.33±0.02	87±8.6	94.1±9.1	9.8±4.9	12.5±6.6	2.2±1.2	2.9±1.6	-	-
Jamilian 2018	3.0±1.7	4.0±1.5	-	-	-	-	-	-	-	-	-	-
Khani 2017	-	-	-	-	102.76±8.81	105.75±8.83	-	-	-	-	116.02±3.12	125.06±2.91
Mejia-Montilla 2018	-	-	-	-	-	-	16.5±3.4	19.6±4.3	3.3±0.9	3.7±0.7	86.3±18.9	102.6±22.9
Mirmasoumi 2017	3.2±3.9	5.2±2.3	0.34±0.02	0.32±0.02	92.6±14.5	94.3±9.1	10.7±6.2	13.5±4.9	2.4±1.5	3.2±1.2	100.8±64.1	122±75.3
Mohammadi 2012	2.11±0.80	2.08±0.80	-	-	85.4±8.95	92.4±9.92	15.1±2.68	16.4±3.39	3.2±0.8	3.8±1.11	119.13±26.04	120.23±28.52
Nadjarzadeh 2015	-	-	-	-	-	-	-	-	-	-	-	-
Rahmani 2016	-	-	-	-	-	-	-	-	-	-	100.6±54	128.3±72.6
Talari 2018	2.5±1.7	2.3±0.8	-	-	-	-	-	-	-	-	-	-

Table S2. Continued

Study	TC (mg/dL)		LDL-C (mg/dL)		HDL-C (mg/dL)		VLDL-C (mg/dL)	
	Intervention	Control	Intervention	Control	Intervention	Control	Intervention	Control
Amini 2018	165.9±26.7	167.3±28.4	93.3±23	94.2±25.3	49.9±9.3	52.1±10.6	22.7±11.4	21.0±9.6
Cussons 2009	180.77±41.15	181.15±38.08	108.96±32.08	106.67±34.78	53.46±12.69	51.54±11.54	-	-
Ebrahimi 2017	-	-	-	-	-	-	-	-
Jamilian 2018	-	-	-	-	-	-	-	-
Khani 2017	180.34±6.33	189.56±5.92	107.79±1.68	117.4±1.57	47.2±1.37	41.56±1.33	-	-
Mejia-Montilla 2018	154.6±17.6	176.1±20.8	84.7±11.2	110.4±15.4	52.7±6.7	47.8±5.6	-	-
Mirmasoumi 2017	166.4±45.9	171.1±33.8	92.5±43.5	92.9±27.8	53.8±8.3	53.8±12.5	20.1±12.8	24.4±15.1
Mohammadi 2012	170.33±32.03	186.63±25.89	102.25±29.59	117.25±27.44	45.86±6.53	45.33±4.49	-	-
Nadjarzadeh 2015	-	-	-	-	-	-	-	-
Rahmani 2016	161.5±31.4	178.6±29.9	94.4±29.8	104.8±26.3	47±9.5	48.1±9.3	20.1±10.8	25.7±14.5
Talari 2018	-	-	-	-	-	-	-	-

Abbreviations: BW, body weight; BMI, body mass index; DHA, docosahexaenoic acid; EPA, eicosapentaenoic acid; FPG, fasting plasma glucose; FINS, fasting insulin; HDL-C, high-density lipoprotein cholesterol; HOMA-IR, homeostatic model of assessment for insulin resistance; hs-CRP, high sensitivity C-reactive protein; LDL-C, low-density lipoprotein cholesterol; n-3 PUFA, n-3 polyunsaturated fatty acid; QUICK, quantitative insulin sensitivity check index; TC, total cholesterol; TG, triglycerides; VLDL-C: very low density lipoprotein-cholesterol.
