# Supplementary figures and images for: Effects of n-3 polyunsaturated fatty acid on metabolic status in women with polycystic ovary syndrome: a meta-analysis of randomized controlled trials
Source: J Ovarian Res. 2023 Mar 17;16:54. doi: 10.1186/s13048-023-01130-4 (PMC10022207; doi:10.1186/s13048-023-01130-4)

**Fig.S1.** Forest plots of HDL-C (a) and LDL-C (b) levels after sensitivity analysis among PCOS patients

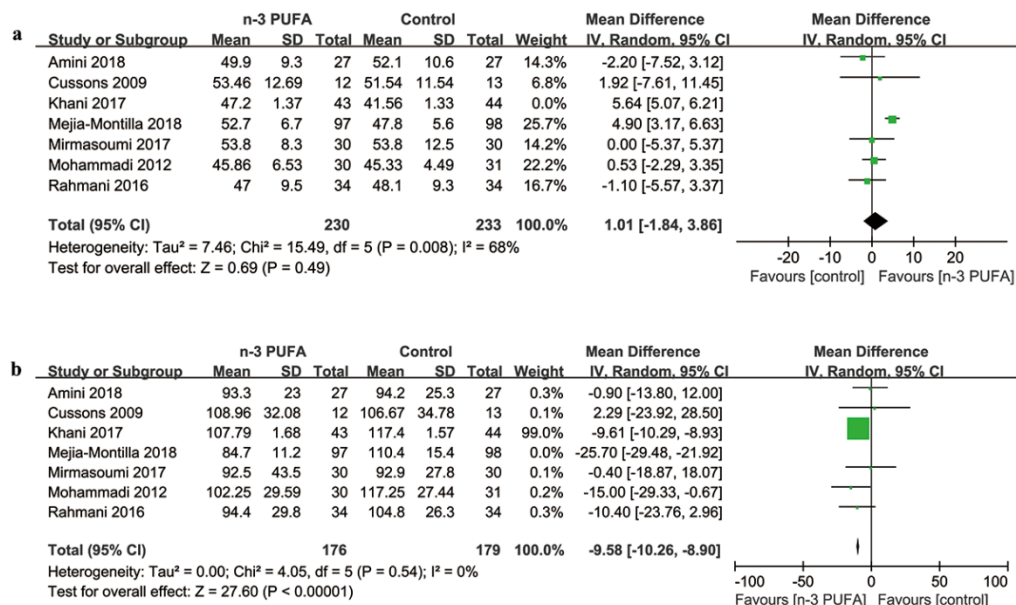

Supplement: Supplementary file 2 — Additional file 2: Supplementary Figure. [file 13048_2023_1130_MOESM2_ESM.pdf]
